# Supplementary material for: Mobilisation of deep crustal sulfide melts as a first order control on upper lithospheric metallogeny
Source: Nat Commun. 2022 Jan 31;13:573. doi: 10.1038/s41467-022-28275-y (PMC8803977; doi:10.1038/s41467-022-28275-y)
Supplement: Supplementary file 1 — Description of Additional Supplementary Files [file 41467_2022_28275_MOESM1_ESM.pdf]

Description of Additional Supplementary Files

Supplementary Data 1. Bulk rock geochemistry for samples from Isola and Sella Bassa.

Supplementary Data 2. Summary of laser ablation ICP-MS analysis of sulfides.

Supplementary Data 3. Quantitative mineralogical data.

Supplementary Data 4. AlphaMELTS modelling methodology and results.
